# Blood Pressure Measurements with Different Currently Available Methods in Elderly Hypertensive Hospitalized Patients: A Real World Cross-Sectional Study

**DOI:** 10.1155/2019/6274545

**Published:** 2019-04-01

**Authors:** Rosaria Del Giorno, Pascal Simon Heiniger, Lorenzo Balestra, Luca Gabutti

**Affiliations:** ^1^Department of Internal Medicine and Nephrology, Regional Hospital of Bellinzona and Valli, Bellinzona, Switzerland; ^2^Internal Medicine Service, La Carità Hospital, Locarno, Switzerland; ^3^Institute of Biomedicine, University of Southern Switzerland, Lugano, Switzerland

## Abstract

**Background:**

The reliability of blood pressure (BP) measurement in hospitalized patients is a topic of debate and the therapeutic implication of the routinely collected BP profiles is probably overestimated. When measurements are performed in elderly patients, further potential sources of misinterpretation occur.

**Methods:**

We conducted a subanalysis of a previous study including 79 over 80-year-old hypertensive patients, hospitalized in an internal medicine ward. Five modalities of BP evaluations (measurement by physicians and nurses, self-measurement by patients, Finometer® beat-to-beat finger monitoring, and 24h monitoring) were analyzed, considering agreement and accuracy.

**Results:**

The mean (SD) age of the patients was 86.9±4.9 years (50% women). Patients' self-measurements of both systolic and diastolic BP (SBP and DBP) did not differ significantly from daytime 24-hour monitoring (D24hBPM) (mean difference -1.52, SE 1.71; p: ns and -0.58, SE 1.19 mmHg; p: ns). Conversely, SBP and DBP registered by nurses did significantly differ (mean difference -7.34, SE 1.42; p=0.007 and -4.7, SE 1.05 mmHg; p=0.003). SBP and DBP measured by patients also showed the better concordance, with lowest biases, and narrowest limits of agreements (LoA) and for SBP higher Kappa statistic values (bias 1.5, LoA -28.9 to 31.9; *κ* 0.563 and bias 0.6, LoA -20.4 to 21.5 mmHg; *κ* 0.412). The patients' sensitivity and specificity in predicting hypertensive systolic D24hBPM were 84.8% and 69.7%, respectively.

**Conclusions:**

In elderly hospitalized patients an alternative to 24hBPM, self-measurements by patients offer the better agreement and reliability in detecting hypertensive values.

## 1. Background

The reliability of blood pressure (BP) measurement in hospitalized patients is a topic of debate and the clinical implication of the routinely collected BP profiles, outside the intensive care units, is not well defined [1, 2]. Several factors, in fact, could influence the in-hospital BP assessment accuracy, ranging from stress generating circumstances to patient-specific, potentially drug enhanced, biological mechanisms [3, 4]. The hemodynamic consequences of stress are overall well known and the tendency of BP to increase, when measured in the presence of a clinician (white-coat effect), was documented in the hospital setting too [5]. This phenomenon could reach a rate of about one-third of patients in a general ward setting and more than half during a preoperative anaesthesia assessment [6, 7].

Further critical points should also be considered in the evaluation of BP profiles in hospitalized patients: the frequent lack of standardized operational protocols to measure BP and the related inconstant quality of the BP measurements performed and the potential lack of accuracy of some of the BP measuring devices [8–11]. Furthermore, in elderly individuals, common comorbidities (e.g., malnutrition, dehydration, and heart failure) associated with biological age-related alterations could affect the BP measurements reproducibility [12]. In the same group of patients, vascular aging, with the associated progression in arterial stiffness, could increase systolic and pulse pressures, leading to isolated systolic hypertension with elevated peripheral but not central values [13]. Moreover, an increased prevalence of white-coat effect, masked hypertension, and abnormal blood pressure variability was documented in the elderly [14]. Considering the listed potential confounding factors, a previous study investigated the reliability of different measurement strategies in the same age frame, analysing in particular differences related to technical aspects such as the professional category of the operator (physician, nurse), the type of device, and the patient posture [15].

In patients over 70 years old, it has been shown that more than 90% of the patients were successful at monitoring BP at home [16]. It has also been demonstrated that in elderly patients affected by hypertension and kidney failure, self-blood pressure measurements (sBPM) at home resulted in a significant improvement of BP control over time [17]. Hence, it is currently, generally, accepted that, in the elderly, BP measurements and therefore BP therapeutic adjustments should be performed on the basis of sBPM, and/or ABPM [18, 19].

Furthermore, BP profiles obtained by self-measurements better correlate with the presence of hypertension target organ damage and with the related cardiovascular risk, compared to office and automated ambulatory monitoring [20–22]. The same was true in large studies which indicate self-BP measurements at home are able to detect morning hypertension and are associated with left ventricular hypertrophy and with BNP levels [23]. Findings about the prognostic value of self-BP monitoring indicate moreover that it is a reliable diagnostic test and an alternative to 24hBPM for decision-making in the management of hypertension [24].

Compared with the large amount of data referring to the out-patient setting, at present, studies exploring the reliability and the best method to assess BP in elderly hospitalized patients are not available. However, on one hand, the elderly population is the most prevalent in the hospital wards, and on the other hand during the hospital stay the home drug therapy is often reassessed and readapted on the basis of the in-hospital evaluation [25]. Hence, the necessity in this fragile subgroup of patients in which inadequate treatment could produce dangerous side effects is to optimize both the measurement strategies and the interpretation of the results.

To contribute in filling the knowledge gap, in the present study we set out to investigate the overall agreement and the clinical accuracy of 5 different types of BP measurement modalities in very old hospitalized patients.

## 2. Methods

### 2.1. Study Design, Participants, and Study Procedures

The present study is the result of a subanalysis of a previous cross-sectional study conducted at the internal medicine service of the teaching hospital “La Carità” in Locarno, Switzerland, from June 2014 to March 2015, in elderly hospitalized patients. The study was approved by the Swiss Ethics Committee and performed in accordance with the Declaration of Helsinki. All study participants provided signed informed consent. Only over 75-year-old patients hospitalized in the internal medicine service, able to understand and sign the informed consent and to perform self-BP measurement, with an automated device operating an arm cuff, were enrolled.

**Figure 1:**
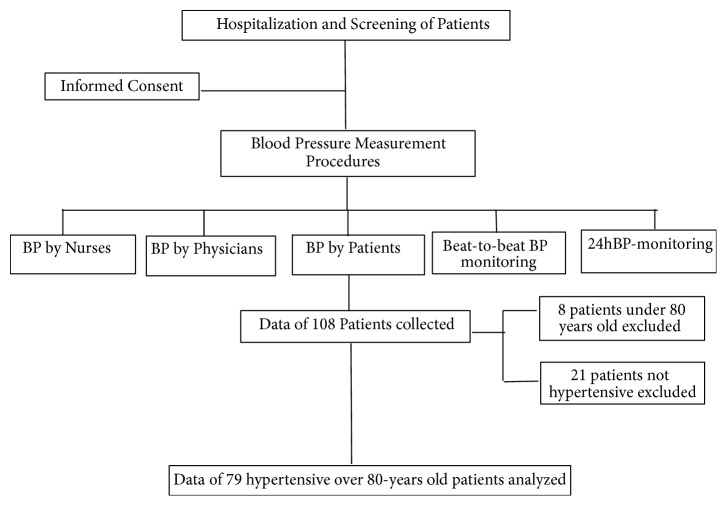
Flow diagram of the study.

From a sample population of 108 participants, data from 79 hypertensive, over 80-year-old patients were included in this analysis. Eight patients less than 80 years old and twenty-one not affected by hypertension were excluded (see Figure 1, flow diagram of the study).

The primary objective of the present study was to evaluate the overall agreement and the clinical accuracy of different blood pressure measurement methods, applied to the selected population.

During the hospital stay, the research team collected information for each participant on sociodemographic characteristics, medical history, chronic diseases, and medication use. Blood pressure measurements, with 5 different methods, were then collected: (i) BP measurement by staff nurses and (ii) by staff physicians, (iii) self BP measurements by patients, (iv) noninvasive continuous finger beat-to-beat BP monitoring, and (v) 24-h BP monitoring (24hBPM).

Blood pressure measurements performed by physicians and nurses and self-measurements by the patients were taken with validated automatic oscillometric devices (506N® monitor, Criticare System Inc., USA) on the nondominant arm. Preliminarily, a circumference measurement of the arm was performed, to determine the size of the cuff to be applied (small <24, medium 24-34, large >34 cm). BP measurements were performed daytime and at the occasion of every visit, BP was collected thrice (at 5-minute intervals). BP measurements taken during the first two days of hospitalization were not used for the statistical analyses.

For the self-BP measurements, all participants were empowered through a short training with a resident physician, and a standardized procedure was applied. Patients were instructed to rest at least 5 minutes before starting the BP readings, avoiding activities which could influence BP (smoking, caffeine, exercise, and eating) in the previous 30 minutes. Patients were also instructed to wear the cuff on the nondominant arm resting on a table at the heart level, and to measure BP, with uncrossed legs, in the sitting position. BP values were registered in a dedicated logbook.

Blood pressure was also evaluated using a noninvasive finger monitor (Finometer®). The device collects a beat-to-beat signal using a small finger cuff applied to the fourth finger of the nondominant hand. The calculated BP value is calibrated for every individual patient at the beginning of the measurements, using a traditional arm cuff incorporated in the device. Following a standard protocol, BP values were recorded 5 times: at 30 seconds and at 1, 2, 4, and 6 minutes. Finally, 24-hour BP monitoring (24hBPM) was performed with a Mobil-O-Graph® device (I.E.M. GmbH, Stolberg, Germany). The cuff was fixed to the nondominant arm and the device was set to obtain automatic readings every 30 minutes during the day (06:00–22:00) and every hour at night (22:00–06:00).

### 2.2. Statistical Analysis

Continuous and categorical variables were expressed as mean (±SD) and percentages, respectively. To test the agreement between daytime 24hBPM (taken as reference) and each BP measurement method under analysis, individual comparisons were performed. Mean differences and standard errors were compared using a parametric method. Bland & Altman plots (plots of the differences between measurements against the mean), biases, limits of agreement (LoA), and rank correlations for each method were graphically and/or numerically shown.

Scatter plots depicting correlations, linear regression, and r^2^ between BP values obtained with the different chosen methods and daytime 24hBMP were also represented.

To analyse the diagnostic agreement between the methods under study we calculated the sensitivity, the specificity, and the negative and positive predictive value, in identifying patients with SBP and/or DBP values above the thresholds. A statistical analysis for assessing the agreement between two clinical evaluation methods (kappa statistic, *κ*) was also performed (*κ* values of 0.4–0.6, 0.6–0.8, and 0.8–1.0 indicate moderate, substantial, and almost perfect agreement respectively).

Statistical analyses were performed using Stata® Statistical Software: Release 15 (STATA Corp LP) and SPSS® 20 (SPSS Inc., Chicago, IL).* p* values <0.05 (two-tailed) were considered significant.

## 3. Results

Table 1 shows baseline demographic and clinical characteristics of the study population. Data of 79 patients were analysed. Mean ± SD age was 86.9 ± 4.9 years. Gender was equally distributed (females 50%); the BMI was 26.8 ± 5.9 kg/m^2^ and the arterial stiffness 12.9 ± 1.2 m/sec. 56.3% of the population were dyslipidemic, 25% were smokers, and 46.3% had a previous cardiovascular event.

Patients' self-measurements of both systolic (SBP) and diastolic (DBP) BP did not differ significantly from D24hBPM (mean difference -1.52, SE 1.71; p: ns and -0.58, SE 1.19 mmHg; p: ns). Conversely, SBP and DBP registered by nurses did significantly differ (mean difference -7.34, SE 1.42; p=0.007 and -4.7, SE 1.05 mmHg; p=0.003) (Table 2).

**Figure 2:**
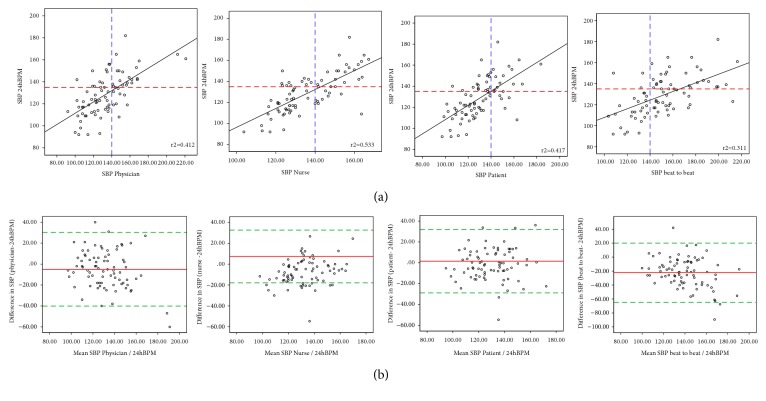
Scatter (a) and Bland & Altman (b) plots comparing systolic blood pressure measured with the proposed strategies (by physicians, nurses, and patients and with a finger beat-to-beat monitor) and daytime systolic 24hBPM. Dashed red lines indicate the high daytime systolic pressure cut-off for 24hBPM; dashed blue lines indicate the high systolic pressure cut-off for the different measurement strategies (physicians: a and b1; nurses: a and b2; patients: a and b3; finger beat-to-beat: a and b4). Solid black lines indicate the linear correlation between systolic blood pressure measured with the different methods and daytime 24hBPM (the r^2^ coefficient is superimposed on the graph). Red lines on the Bland-Altman plot indicate the bias; dotted green lines indicate the limits of agreement.

**Figure 3:**
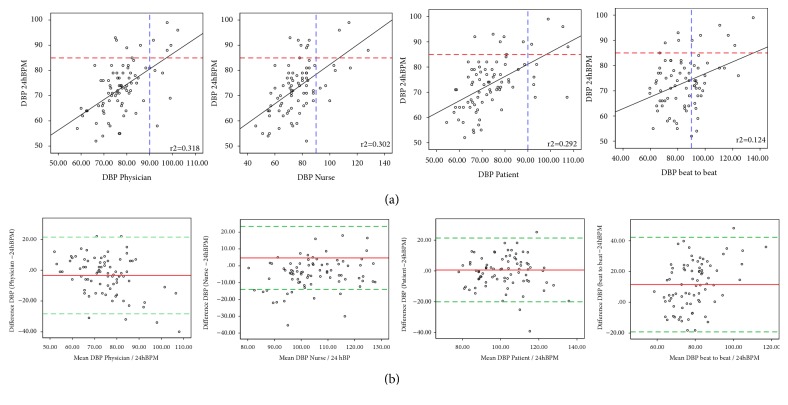
Scatter (a) and Bland & Altman (b) plots comparing diastolic blood pressure measured with the proposed strategies (by physicians, nurses, and patients and with a finger beat-to-beat monitor) and daytime diastolic 24hBPM. Dashed red lines indicate the high daytime diastolic pressure cut-off for 24hBPM; dashed blue lines indicate the high diastolic pressure cut-off for the different measurement strategies (physicians: a and b1; nurses: a and b2; patients: a and b3; finger beat-to-beat: a and b4). Solid black lines indicate the linear correlation between diastolic blood pressure measured with the different methods and daytime 24hBPM (the r^2^ coefficient is superimposed on the graph). Red lines on the Bland-Altman plot indicate the bias; dotted green lines indicate the limits of agreement.

Overall agreement for SBP and DBP comparing the different methods with the D24hBPM are illustrated using scatter plots in the top panels and Bland & Altman plots in the bottom panels of Figures 2 and 3, which graphically summarize data shown in Table 2. In the Bland & Altman plots biases and limits of agreement (LoA) are also shown.

The highest concordance, with lowest biases and narrowest limits of agreement, was found for SBP and DBP measured by patients compared with D24hBPM (bias 1.5, LoA -28.9 to 31.9 and bias 0.6, LoA -20.4 to 21.5 mmHg) (Table 2, Figures 2(b) and 3(b)).

Self-BP measurements also presented a high rank correlation (r=0.645 for SBP and 0.540 for DBP) (Table 2), with a cut-off point showing that patients are reasonably sensitive and specific in detecting high blood pressure (Figures 2(a) and 3(a)).

Systolic and diastolic BP readings made by nurses exceeded the mean D24hBPM with, respectively, a bias of 7.3 (LoA -17.9 to 32.6) and 4.7 mmHg (LoA -14.0 to 23.4).

The difference between readings by physicians and 24hBPM, decreased as the blood pressure increased.

SBP measured by physicians and daytime 24hBPM presented high rank correlation (r=0.642) with a cut-off point showing that physicians are sensitive in detecting high blood pressure, but poorly specific (Figure 2(a)). Physicians' Bland & Altman biases for SBP and DBP were, respectively, -5.1 and -3.4 mmHg with LoA of -40.1 to 30.3 and -28.3 to 21.5 mmHg (Figures 2(b) and 3(b)).

Systolic and diastolic BP measurements made by finger beat-to-beat on average exceeded mean D24hBPM with, respectively, a bias of 22.2 (LoA -20.3 to 64.9 mmHg) and 11.5 (-19.2 to 42.24) mmHg.

In predicting high systolic daytime 24hBPM (SBP**>**135mm Hg) all methods showed a specificity > 80% and a sensitivity > 60% (Table 3).

Systolic BP measurements, performed by patients, presented the highest sensitivity and specificity (84.8% and 69.7%, respectively) in predicting hypertensive daytime 24hBPM values. Moreover, self-systolic measurements by patients presented the better agreement estimated by K statistic with a *κ* value of 0.563, allowed the highest rate of correct classification of the BP pattern (78.5%), and showed the lowest rate of false positive and false negative ( 23.3 and 20.4%, respectively) (Table 3). Diastolic BP readings performed by patients showed also a good agreement with daytime 24hBPM with 89.9% of correct classification (Table 3).

In detecting combined high 24-hBPM SBP and DBP the finger beat-to-beat method showed the better values in terms of sensitivity (98.5%) and false positive rate (25.0%). Overall, Finometer® performed better in correctly classifying patients with high SBP and DBP (89.8%). This effect was mainly due to the capacity of Finometer® to detect 100% of the cases of high DBP.

An agreement analysis between BP measurement methods was also performed in the subgroup of patients with diabetes (26 out of 79). Results showed a similar trend, with the best agreement, for both SBP and DBP, between patients' self-BP measurements and 24hBPM (the k statistic values obtained by physicians, nurses, patients, and finger beat-to-beat compared with 24hBPM were, respectively, 0.461, 0.456, 0.698, and 0.442 for SBP; and 0.284, 0.345, 0.623, and 0.344 for DBP). However the small sample size did not allow definitively concluding that the pattern concordance of the two subpopulations (diabetics vs. nondiabetics) was the same.

## 4. Discussion

The correct way of interpreting blood pressure measurements in hospitalized patients outside the intensive care setting is still a matter of debate. Furthermore, concerns exist when evaluation of BP is performed in elderly patients due mainly to a higher prevalence of pathological arterial wall changes with increased arterial stiffness, masked hypertension, extreme BP variability, and decompensated baroreceptor function [26–28]. In the present study we investigated the accuracy and the agreement of currently available measurement methods in very old (> 80 years) hypertensive patients admitted in an internal medicine service of a teaching hospital. Data from previous studies suggest that ambulatory blood pressure monitoring and home self-blood pressure measurements are likely to be the most accurate BP assessment strategies for elderly subjects [29, 30].

Our findings boost the evidence that, also in the hospital setting, in the subgroup of over 80-year-old patients, the self-measurement of BP represents a reliable alternative to a 24-hour BP monitoring.

The high quality concordance with lower bias and limits of agreements, compared with physician and nurse measurements, also considering the previous data from the literature [25, 26], suggests that the same conclusions could be valid for other in-hospital adult age groups.

Our study also corroborates the widely accepted concept that measurements of BP performed by nurses and physicians in the hospital setting are inaccurate if compared with the results of a daytime 24-hour monitoring [31]. The larger positive bias obtained measuring systolic BP with the beat-to-beat was probably generated by the technology used, exposing patients to an extra stress, related to the complexity of the device and the measurement modality chosen. Surprisingly, in our study BP assessed by physicians presented a better agreement with daytime 24hBPM then measurements performed by nurses. This finding could be the consequence of the organisation of the in-hospital health care delivery system, exposing patients to a larger number of nurses compared to physicians, hence increasing the risk and the related stress of being seen by an unknown person. Nevertheless measurements obtained by both professional categories significantly over- and underestimate systolic and diastolic BP values in, respectively, about half and one-third of the cases. These findings raise relevant clinical concerns considering the short-and long-term potential consequences of inadequate in-hospital BP assessment. Inappropriate therapeutical management induced by overestimation of blood pressure could in fact lead to orthostatic symptoms with dizziness, thereby increasing the risk of falls and fractures [32, 33].

The message is not new but interesting if seen in the context of the study. Only a few previous analyses focused in fact on blood pressure behaviour in the subgroup of over 80-year-old patients [34]. The features that make this study unique, apart from the age frame of the patients, are the in-hospital real world setting, the exhaustive spectrum of BP measurement strategies applied, and the in depth statistical analysis.

However, we have to address some limitations of our study. The present evaluation is a subanalysis of a previous cross-sectional study and the agreement assessment in hypertensive over 80-year-old patients was not the objective of the initial investigation. Moreover, the sample size was small and the cut­off points of systolic and diastolic blood pressure to define hypertensive values in elderly are still widely debated. For the statistical analysis, as defined by previous recommendations for hypertensive patients, we chose, irrespective of age, 140/90 and 135/85 mmHg as the upper limit, for clinical readings (physicians, nurses, and patients) and 24hBPM, respectively [35].

In conclusion, in our study, in over 80-year-old in-hospital patients, using the daytime 24-hour monitoring as standard, self-blood pressure measurement was the most accurate assessment method, compared with the alternative modalities tested. Blood pressure readings performed not only by nurses but also by physicians significantly over-/underestimate the true pressure profile exposing patients to inadequate and potentially dangerous therapeutic interventions. Individual blood pressure values are instantaneous expressions of a complex hemodynamic context and they cannot be interpreted without an extensive knowledge of the patient and of the underling mechanisms.

## Figures and Tables

**Table 1:** Characteristics of the Study Population.

Age, years	86.9 ± 4.9
Gender, females	40 (50)
BMI, Kg/m^2^	26.8 ± 5.9
Diabetes	26 (32.5)
Dyslipidemia	45 (56.3)
Current Smoking	20 (25)
Creatinine, mmol/L	98.9±60.3
GFR, mL/min/1.73 m^2^	50.6 ± 25.9
CVD	37 (46.3)
PWV, m/sec	12.9 ± 1.2
24hBPM daytime, SBP mmHg	128.9 ± 10.5
24hBPM daytime, DBP mmHg	73.3±10.3
Nurse SBP mmHg	135.1± 14.1
Nurse DBP mmHg	77.4 ±8.9
Patient Self SBP mmHg	130.4±17.5
Patient Self DBP mmHg	73.9 ±11.4
Physician SBP mmHg	134.0 ± 23.0
Physician DBP mmHg	76.7 ± 14.9
Beat-to-beat, SBP mmHg	151.1 ± 25.1
Beat-to-beat, DBP mmHg	84.8±15.5

Data are expressed as mean ± (SD) or as absolute (n) and relative (%) frequencies.

BMI: Body Mass Index; GFR: Glomerular Filtration Rate; CVD: Cardiovascular Diseases; PWV: Pulse Wave Velocity; 24hBPM: 24 hour Blood Pressure Monitoring; SBP: Systolic Blood Pressure; DBP: Diastolic Blood Pressure.

**Table 2:** Overall agreement of systolic (SBP) and diastolic (DBP) blood pressures comparing the tested measurement methods with mean daytime 24hBPM (mean difference, rank correlation, limits of agreement, and rank correlation from the Bland & Altman plot).

Method	SBP±SD	Mean Difference	Standard Error	*p* value	Correlation R^2^	*p* value	Bias Limits of Agreement 95%	Bland& Altman Plot *r*	*p* value
Physician vs. 24hBPM	134.0 ± 23.1vs 128.9 ± 18.5	-5.13	2.03	0.125	0.642*∗*	0.001*∗*	-5.1 (-40.1 to 30.3)	-0.28	0.011*∗*
Nurse vs. 24hBPM	136.2 ± 15.1vs 128.9 ± 18.5	-7.34	1.42	0.007*∗*	0.734*∗*	0.001*∗*	7.34 ( -17.9 to 32.6)	-0.28	0.019*∗*
Patient vs. 24hBPM	130.4±17.6 vs 128.9 ± 18.5	-1.52	1.71	0.596	0.645*∗*	0.001*∗*	1.5 (-28.9 to 31.9)	0.06	0.573
Beat-to-beat vs. 24hBPM	151.1 ± 25.1 vs 128.9 ± 18.5	-22.27	2.39	<0.001*∗*	0.557*∗*	0.001*∗*	22.2 (-64.9 to 20.3)	0.35	0.002*∗*

Method	DBP±SD	Mean Difference	Standard Error	*p* value	Correlation R^2^	*p* value	Limits of Agreement 95%	Bland& Altman Plot *r*	p-value

physician vs. 24hBPM	76.7 ± 14.9 vs 73.3 ± 10.3	-3.4	1.40	0.096	0.564*∗*	0.001*∗*	-3.4 (-28.3 to 21.5)	-0.42	<0.001*∗*
nurse vs. 24hBPM	77.9 ± 9.3 vs 73.3 ± 10.3	-4.7	1.05	0.003*∗*	0.549*∗*	0.001*∗*	4.7 (-14.0 to 23.4)	-0.12	0.307
patient vs. 24hBPM	73.8 ± 11.4 vs 73.3 ± 10.3	-0.58	1.19	0.737	0.540*∗*	0.001*∗*	0.6 (-20.4 to 21.5)	0.13	0.252
Beat-to-beat vs. 24hBPM	84.8 ± 15.5 vs 73.3 ± 10.3	-11.5	1.72	<0.001*∗*	0.351*∗*	0.001*∗*	11.5 (-19.2 to 42.24)	0.41	0.002*∗*

24hBPM: 24 h blood pressure monitoring; BP: blood pressure; DBP: diastolic blood pressure; SBP: systolic blood pressure; SD: standard deviation.

*Notes.∗*p<0.05; BP expressed in mmHg

**Table 3:** Diagnostic agreement s, specificity, and likelihood ratios) between different measures (nurse, doctor, self-measurement, beat-to-beat) in predicting ambulatory blood pressure in hospital setting.

High 24-h SBP-ABPM (>135mm Hg)	Sensitivity (%)	Specificity (%)	Positive Predictive Values (%)	Negative Predictive Values (%)	Correctly classified (%)	False Positive (%)	False Negative (%)	K statistic
Physician	63.6	82.6	72.4	67.5	74.7	27.6	24.0	0.428
Nurse	66.7	82.6	73.3	77.5	75.9	26.7	22.4	0.496
Patient	69.7	84.8	76.7	79.6	78.5	23.3	20.4	0.563
Beat –to-beat- Finometer	45.5	84.8	68.2	68.4	68.4	31.8	31.5	0.404

High 24-h diastolic blood pressure ABPM(>85mm Hg)								

Physician	20.0	98.5	66.7	89.4	88.6	33.3	10.3	0.228
Nurse	40.0	97.1	66.7	91.8	89.8	33.3	8.2	0.451
Patient	30.0	98.5	75.0	90.7	89.9	25.0	9.3	0.412
Beat-to-beat Finometer	10.0	100.0	100.0	88.5	88.61	0	11.5	0.163

High 24-h SBP/DBP -ABPM								

Physician	10.0	95.5	50.0	88.3	87.3	50.0	11.7	0.315
Nurse	40.00	95.5	57.1	91.7	88.6	42.9	8.3	0.466
Patient	30.0	97.10	60.0	90.5	88.6	40.0	9.46	0.441
Beat-to-beat Finometer	30.0	98.5	75.0	90.6	89.8	25.0	9.3	0.223

## Data Availability

The data used to support the findings of this study are available from the corresponding author upon request.

## Abbreviations

BP: Blood pressure
24hBPM: 24-hour blood pressure monitoring
D24hBPM: Daytime 24-hour blood pressure monitoring
LoA: Limit of agreement
SBP: Systolic blood pressure
DBP: Diastolic blood pressure
sBPM: Self-blood pressure measurements.
